# The heterogeneous nuclear ribonucleoprotein hnRNPM inhibits RNA virus-triggered innate immunity by antagonizing RNA sensing of RIG-I-like receptors

**DOI:** 10.1371/journal.ppat.1007983

**Published:** 2019-08-21

**Authors:** Pan Cao, Wei-Wei Luo, Chen Li, Zhen Tong, Zhou-Qin Zheng, Lu Zhou, Yong Xiong, Shu Li

**Affiliations:** 1 Department of Infectious Diseases, Zhongnan Hospital of Wuhan University, College of Life Sciences, Medical Research Institute, Wuhan University, Wuhan, China; 2 Wuhan Institute of Virology, Chinese Academy of Sciences, Wuhan, China; Duke University Medical Center, UNITED STATES

## Abstract

Recognition of viral RNA by the retinoic acid-inducible gene-I (RIG-I)-like receptors (RLRs), including RIG-I and MDA5, initiates innate antiviral responses. Although regulation of RLR-mediated signal transduction has been extensively investigated, how the recognition of viral RNA by RLRs is regulated remains enigmatic. In this study, we identified heterogeneous nuclear ribonucleoprotein M (hnRNPM) as a negative regulator of RLR-mediated signaling. Overexpression of hnRNPM markedly inhibited RNA virus-triggered innate immune responses. Conversely, hnRNPM-deficiency increased viral RNA-triggered innate immune responses and inhibited replication of RNA viruses. Viral infection caused translocation of hnRNPM from the nucleus to the cytoplasm. hnRNPM interacted with RIG-I and MDA5, and impaired the binding of the RLRs to viral RNA, leading to inhibition of innate antiviral response. Our findings suggest that hnRNPM acts as an important decoy for excessive innate antiviral immune response.

## Author summary

Infection by virus, such as the RNA virus Sendai virus, induces the host cells to express proteins that mediate antiviral immune responses. Upon infections, the retinoic acid-inducible gene-I (RIG-I)-like receptors (RLRs) detects the intracellular viral RNA and initiates innate immune responses. Although the regulation of RLR-mediated signal transduction has been extensively investigated, how the recognition of viral RNA by RLRs is regulated remains enigmatic. In this study, we found that a protein called hnRNPM plays an important role in the process of antiviral immune response. hnRNPM does this by impairing the binding of the RLRs to viral RNA. Our results suggest that hnRNPM is an inhibitor of RNA virus-induced signaling which provides a critical control mechanism of viral RNA sensing for the host to avoid excessive and harmful immune response.

## Introduction

Innate immune response provides the first line of host defense against invading microbial pathogens [1]. Upon infection, the conserved microbial components called pathogen-associated molecular patterns (PAMPs) are sensed by cellular pattern recognition receptors (PRRs). This leads to induction of type I interferons (IFNs), pro-inflammatory cytokines, and other downstream effector genes. These downstream effector proteins mediate innate immune and inflammatory responses to inhibit microbial replication and clear infected cells [1, 2].

Viral nucleic acids are major PAMPs that are sensed by the host cells after viral infection. Extracellular viral RNA is recognized by transmembrane and endosomal Toll-like receptor 3 (TLR3), which is expressed mostly in immune cells [3], whereas intracellular viral RNA is detected by the retinoic acid-inducible gene-I (RIG-I)-like receptors (RLRs), including RIG-I and MDA5[4]. Genetic studies have demonstrated that RIG-I and MDA5 play crucial roles in innate immune response to different types of RNA viruses [1] [5]. RIG-I and MDA5 utilize similar signaling pathways to induce downstream antiviral genes. Upon binding to viral RNA, RIG-I or MDA5 undergoes conformational changes and is recruited to the mitochondrial membrane-located adaptor protein VISA (also called MAVS, IPS-1 and Cardif) [6–9]. This triggers the formation of large prion-like VISA polymers, which in turn serve as platforms for recruitment of TRAF2/3/5/6 through its TRAF-binding motifs [10, 11]. The TRAF proteins further recruit TBK1 and the IKK complex to phosphorylate IRF3 and IκBα respectively, leading to activation of IRF3 and NF-κB and induction of downstream antiviral effectors.

Both RIG-I and MDA5 contain two tandem caspase-recruitment domains (CARDs) at their N terminus, which mediate downstream signaling; a central DExD/H helicase domain with an ATP-binding motif; and a C-terminal RNA-binding domain [5]. Although RIG-I and MDA5 share similar signaling features and structural homology, various studies have demonstrated that the two helicases may discriminate among different ligands to trigger innate immune response. It has been demonstrated that RIG-I preferably recognizes viral 5’-ppp double-strand (ds) RNA and relatively short (approximately 300 bp) dsRNA, while MDA5 has higher affinity to long dsRNA [12–14]. Various studies have shown that RIG-I is essential for induction of downstream antiviral effector genes in response to RNA viruses including Sendai virus (SeV), vesicular stomatitis virus (VSV), Newcastle disease virus (NDV), influenza virus and Japanese encephalitis virus (JEV), whereas MDA5 is critical for the detection of picornaviruses, such as encephalomyocarditis virus (EMCV) [15, 16].

RLR-mediated innate antiviral responses are regulated by distinct mechanisms. For examples, TRIM25, TRIM4, Riplet (also known as RNF135), TRIM13, USP4, USP3, USP15, USP21, CKII, PP1α/γ, and TRIM38 have been reported to regulate the post-translational modifications of RLRs [17–27]. MEX3C in stress granules enhances the affinity between viral RNA and RIG-I [28]. RAVER1 regulates MDA5- but not RIG-I-mediated antiviral immune response by promoting the binding of MDA5 to viral RNA [29]. More recently, it has been shown that ZCCHC3 acts as a co-receptor for the binding of RIG-I and MDA5 to viral RNA [30]. However, how RLR activation is monitored to prevent excessive innate antiviral response is unclear.

The heterogeneous nuclear ribonucleoprotein M (hnRNPM) contains three RNA recognition motif (RRM) domains. It has been shown that hnRNPM is involved in pre-mRNA splicing and diverse aspects of RNA metabolism, including translational control, telomere biogenesis, mRNA stability, and trafficking[31, 32]. Here we identified hnRNPM as a decoy of innate antiviral response. Viral infection led to export of hnRNPM from the nucleus to the cytoplasm, at where it impaired the binding of RLRs to viral RNA and subsequent innate antiviral response. Our findings reveal a mechanism on how the sensing of viral RNA by RLRs is properly regulated.

## Results

### hnRNPM inhibits RNA virus-triggered signaling

**Fig 1:**
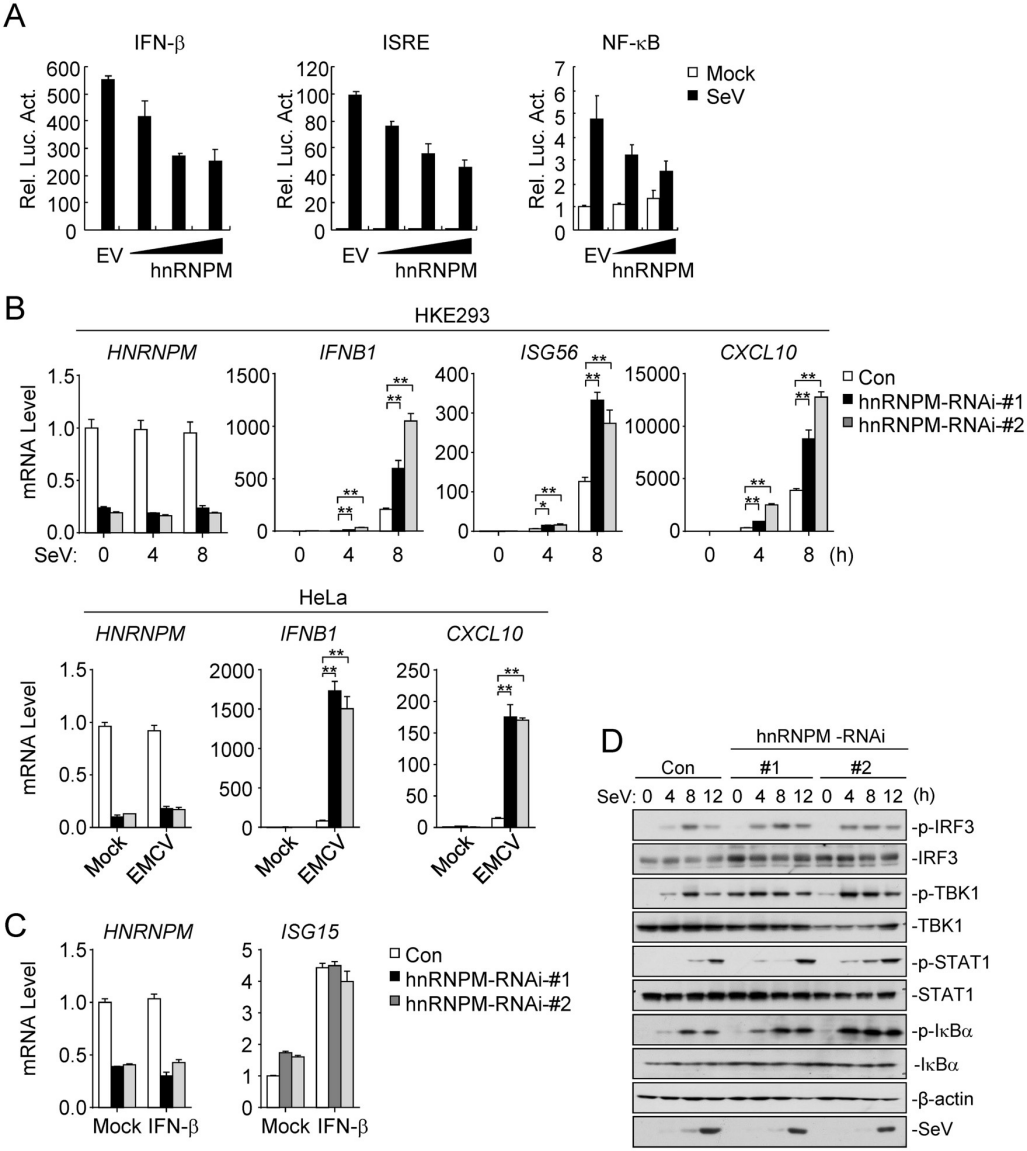
Identification of hnRNPM as an inhibitor of RNA virus-triggered signaling. (A) Effects of hnRNPM on SeV-induced activation of the IFN-β promoter, ISRE and Nifty. HEK293 cells (1×10^5^) were transfected with the IFN-β reporter (50 ng), ISRE (50 ng) and NF-κB (2 ng) and increased amounts of hnRNPM plasmid or empty vector (EV) for 20 h, and then left uninfected or infected with SeV for 12 h before luciferase assays. (B) Effects of hnRNPM knockdown on virus-induced transcriptions of downstream antiviral genes. HEK293 or HeLa cells stably transduced with control shRNA (Con) and hnRNPM-shRNAs were infected with SeV or EMCV for the indicated times before qPCR analysis. *p < 0.05, **p < 0.01 (unpaired t test). (C) Effects of hnRNPM knockdown on IFN-β-induced transcriptions of downstream genes. Control and HEK293 cells stably expressing hnRNPM-shRNAs were treated with IFN-β (100 ng/ml) or left untreated for 6 h before qPCR analysis. (D) Effects of hnRNPM knockdown on SeV-induced phosphorylation of downstream components. Immunoblot analysis of the indicated proteins in HEK293 cells stably expressing control or hnRNPM shRNAs after un-infected or infected with SeV for the indicated times.

To identify candidate molecules involved in viral RNA-triggered innate immune response, we screened ~10,000 independent human cDNA clones for their abilities to regulate IFN-β promoter activity by reporter assays and identified hnRNPM as a candidate protein. As shown in Fig 1A, overexpression of hnRNPM inhibited SeV-triggered activation of the IFN-β promoter, ISRE and NF-κB. Conversely, knockdown of hnRNPM facilitated SeV- and EMCV-induced transcription of the *IFNB1*, *ISG56* and *CXCL10* genes (Fig 1B), but not IFN-β-induced transcription of the *ISG15* gene (Fig 1C). Consistently, knockdown of hnRNPM enhanced SeV-induced phosphorylation of IRF3, TBK1, STAT1 and IκBα (Fig 1D). These results suggest that hnRNPM negatively regulates RNA virus-triggered induction of antiviral genes.

### hnRNPM-deficiency facilitates innate immunity to RNA virus

**Fig 2:**
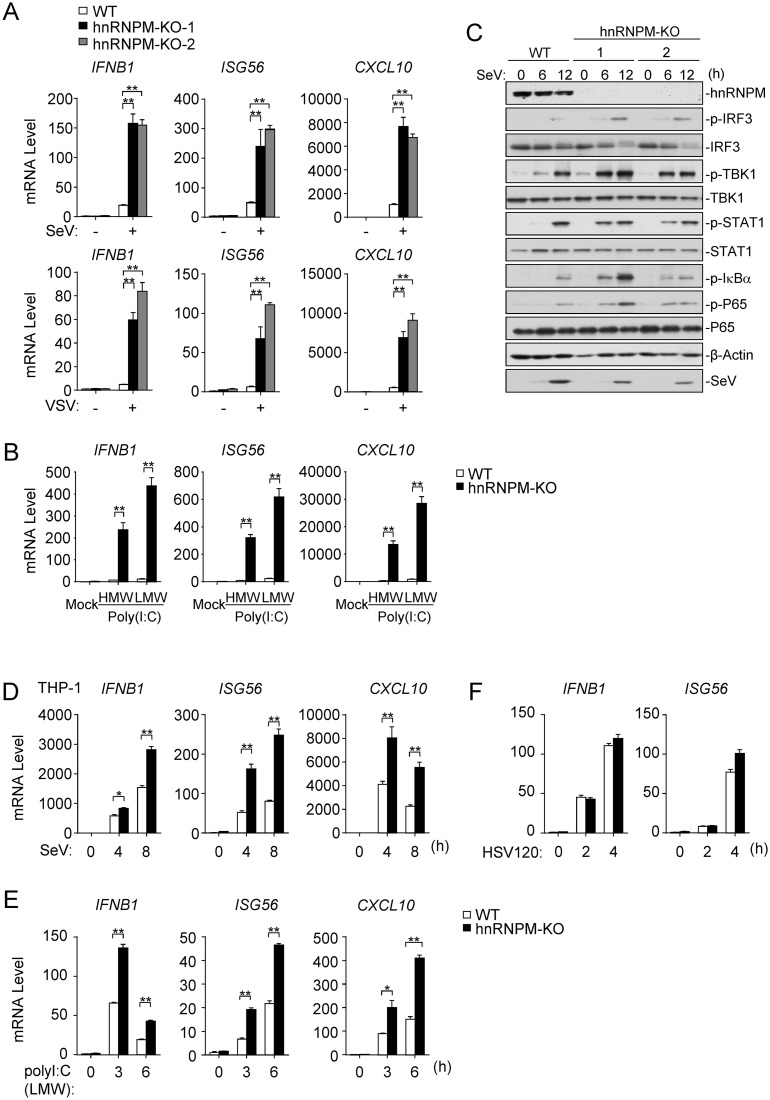
hnRNPM-deficiency facilitates innate immunity to RNA virus. (A) Effects of hnRNPM-deficiency on virus-induced transcriptions of downstream genes. hnRNPM-deficient (KO) HEK293 clones were generated by the CRISPR-Cas9 method. hnRNPM-KO and control HEK293 cells were left un-infected or infected with SeV, VSV for 8 h before qPCR analysis. (B) Effects of hnRNPM-deficiency on transfected poly(I:C)-induced transcriptions of downstream genes. hnRNPM-KO and control HEK293 cells were transfected with high- or low-molecular-weight poly(I:C) for 12 h before qPCR analysis. (C) Effects of hnRNPM-deficiency on SeV-induced phosphorylation of downstream components. hnRNPM-KO and control HEK293 cells were left un-infected or infected with SeV for the indicated times before immunoblotting analysis. (D) Effects of hnRNPM-deficiency on SeV-induced transcriptions of downstream genes in THP-1 cells. hnRNPM-KO and control THP-1 cells were left un-infected or infected with SeV for the indicated times before qPCR analysis. (E) Effects of hnRNPM-deficiency on poly(I:C)-induced transcriptions of downstream genes in THP-1 cells. hnRNPM-KO and control THP-1 cells were transfected with low-molecular-weight poly(I:C) for the indicated times before qPCR analysis. (F) Effects of hnRNPM-deficiency on HSV120-induced transcriptions of downstream genes in THP-1 cells. hnRNPM-KO and control THP-1 cells were transfected with HSV120 for the indicated times before qPCR analysis. *p < 0.05, **p < 0.01 (unpaired t test).

To investigate whether endogenous hnRNPM is required for innate immune response to RNA virus, we generated hnRNPM-deficient HEK293 individual clones by the CRISPR-Cas9 method. We found that transcription of the *IFNB1*, *ISG56*, and *CXCL10* genes induced by SeV or VSV were markedly increased in hnRNPM-deficient cells (Fig 2A). Consistently, transcription of the *IFNB1*, *ISG56*, and *CXCL10* genes induced by cytoplasmic transfected high- or low-molecular-weight (HMW or LMW) poly(I:C) was markedly increased in hnRNPM-deficient cells (Fig 2B). Consistently, phosphorylation of TBK1, IRF3, IκBα, p65 and STAT1 induced by SeV was markedly increased in hnRNPM-deficient cells in comparison to control cells (Fig 2C).

To determine whether the effects of hnRNPM-deficiency are cell type-specific, we generated hnRNPM-deficient THP1 cells. We found that transcription of the *IFNB1*, *ISG56*, and *CXCL10* genes induced by SeV (Fig 2D) and transfected LMW-poly(I:C) (Fig 2E) was markedly increased in hnRNPM-deficient THP1 cells in comparison to control cells. In similar experiments, hnRNPM-deficiency had no marked effects on transcription of these downstream genes induced by transfected DNA mimics such as HSV120 (a synthetic 120-mer dsDNA representing the genomes of HSV-1), HT-DNA (herring testis DNA) and VACV70 (70-mer dsDNA representing the genomes of VACV) (Fig 2F and S1B Fig). Interestingly, we found that knockout of hnRNPM increased transcription of downstream genes induced by DNA virus HSV-1 (S1A Fig), which indicate that hnRNPM regulates HSV-1-triggered innate immune responses in a viral DNA-independent manner. These results suggest that hnRNPM negatively regulates viral RNA- but not DNA-triggered induction of downstream effector genes.

### hnRNPM-deficiency inhibits replication of RNA virus

**Fig 3:**
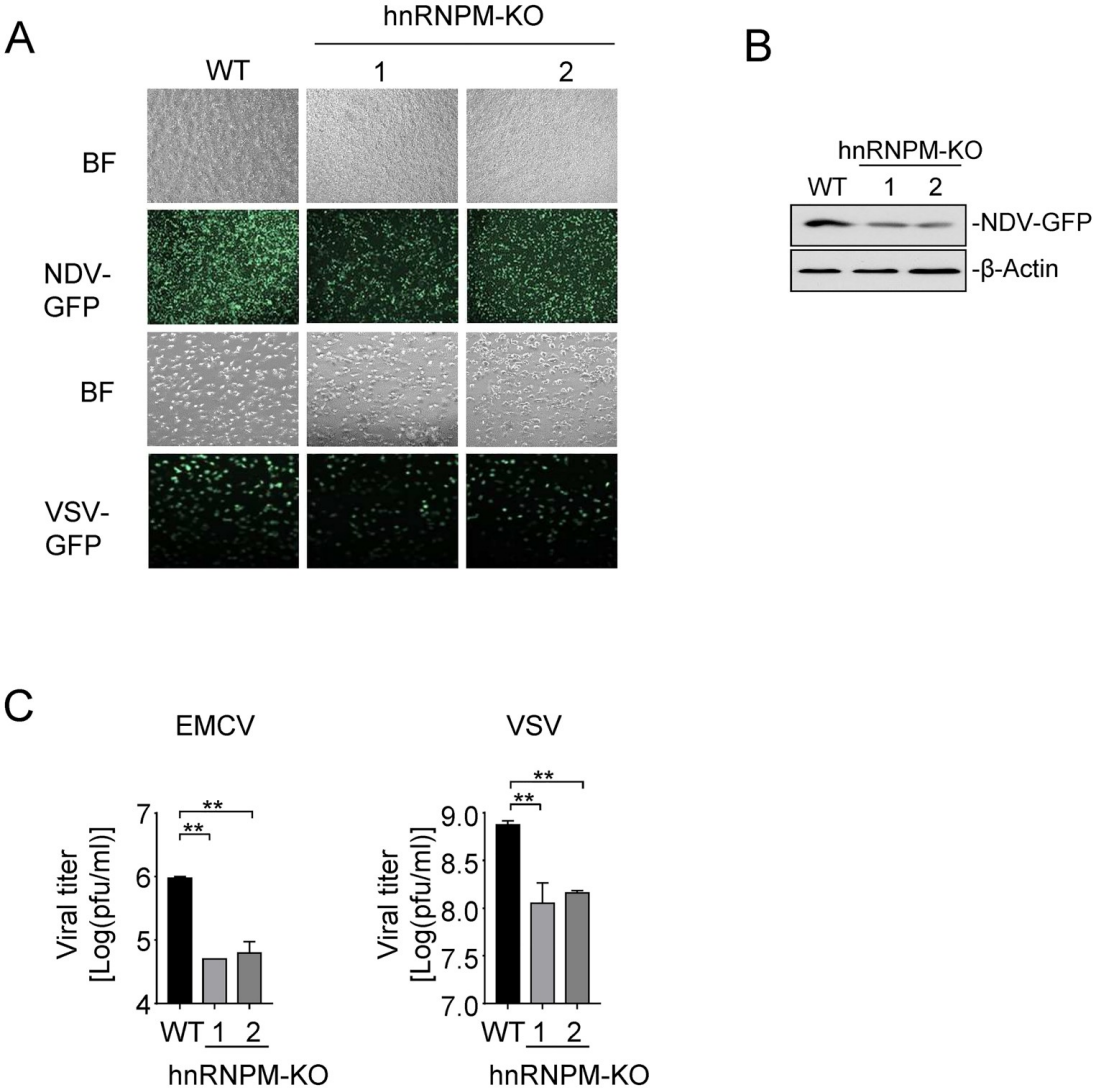
Knockout of hnRNPM inhibits replication of RNA virus. (A) Effects of hnRNPM-deficiency on viral replication. hnRNPM-KO and control HEK293 cells were infected with NDV-GFP (MOI = 1) for 24 h or with VSV-GFP (MOI = 0.1) for 18 h followed by microscopy imaging. MOI, multiplicities of infection; BF, bright field. (B) Immunoblot analysis of NDV replication. (C) Effects of hnRNPM-deficiency on VSV and EMCV replication. hnRNPM-KO and control HEK293 cells were infected with EMCV (MOI = 0.1) or VSV (MOI = 0.01). The supernatants were harvested 36 h after infection and used for standard plaque assays. **p < 0.01 (unpaired t test).

Since hnRNPM inhibits RLR-mediated signaling, we examined the roles of hnRNPM in cellular antiviral response. Previous studies have demonstrated that RIG-I is essential for the antiviral innate response to NDV and VSV, whereas MDA5 is critical for the detection of EMCV. We found that the replication of GFP-tagged NDV and VSV was markedly inhibited in hnRNPM-deficient cells compared with control cells as monitored by GFP expression (Fig 3A & 3B). Plaque assays showed that viral titers of VSV and EMCV were much lower in hnRNPM-deficient cells compared with in control cells (Fig 3C). These results suggest that hnRNPM-deficiency inhibits replication of RNA virus.

### Infection of RNA virus induces export of hnRNPM from the nucleus to cytoplasm

**Fig 4:**
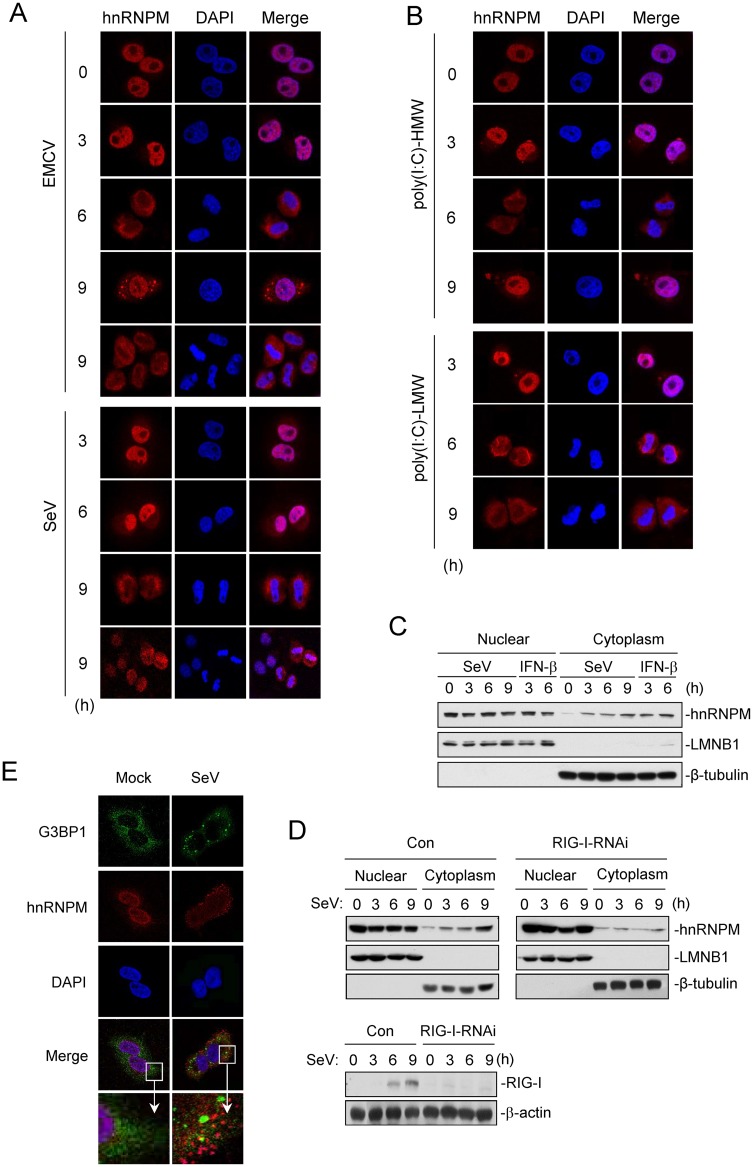
RNA virus induces export of hnRNPM from the nucleus to cytoplasm. (A,B) Immunostaining of hnRNPM (red) in HeLa cells infected with EMCV or SeV (A), or transfected with poly(I:C)-HMW or poly(I:C)-LMW (B) for the indicated times. Scale bars represent 50 μm. (C)Effects of SeV infection on subcellular distribution of hnRNPM. HEK293 cells were treated with SeV and IFN-β for indicated times. The cellular fraction were analyzed by immunoblotting. LMNB1 and Tubulin were used as a nuclear and cytoplasmic marker protein respectively. (D) Effect of knockdown RIG-I on the cellular re-distribution of hnRNPM. HEK293 stably transfected with control shRNA (Con) or RIG-I-shRNAs were infected with SeV for indicated times. Cell were collected for subcellular fraction assay similarly as in (C). (E) Immunostaining of hnRNPM (red) and G3BP1 (green) in HeLa cells infected or un-infected with SeV for 6 h. Scale bars represent 50 μm.

Previous studies have shown that hnRNPM is mainly located in the nucleoplasm and plasma membrane, and barely detected in the cytoplasm [33–35]. We further examined the cellular localizations of hnRNPM before and after viral infection. Confocal microscopy revealed that hnRNPM was mostly localized in the nucleoplasm in uninfected cells. However, SeV or EMCV infection resulted export of hnRNPM from the nucleus to cytoplasm (Fig 4A). Similarly, transfection of poly(I:C) also induced export of hnRNPM from the nucleoplasm to cytoplasm (Fig 4B). Consistent with confocal microscopy, subcellular fractionation analysis showed that hnRNPM was enriched in the cytosol after SeV infection. Notably, recombinant IFN-β also induced hnRNPM translocation (Fig 4C). In addition, the hnRNPM translocation after viral infection was inhibited in RIG-I knockdown cells (Fig 4D). Antiviral stress granules are reported to be a platform for the detection of viruses [36, 37]. Although it seems hnRNPM formed puncta structures in cytoplasm after virus infection, confocal microscopy experiments showed that hnRNPM was not co-localized with G3BP1 (as a marker of stress granules) (Fig 4E). These results suggest that RLR signaling activation causes export of hnRNPM from the nucleus to cytoplasm.

### hnRNPM interacts with RIG-I and MDA5

**Fig 5:**
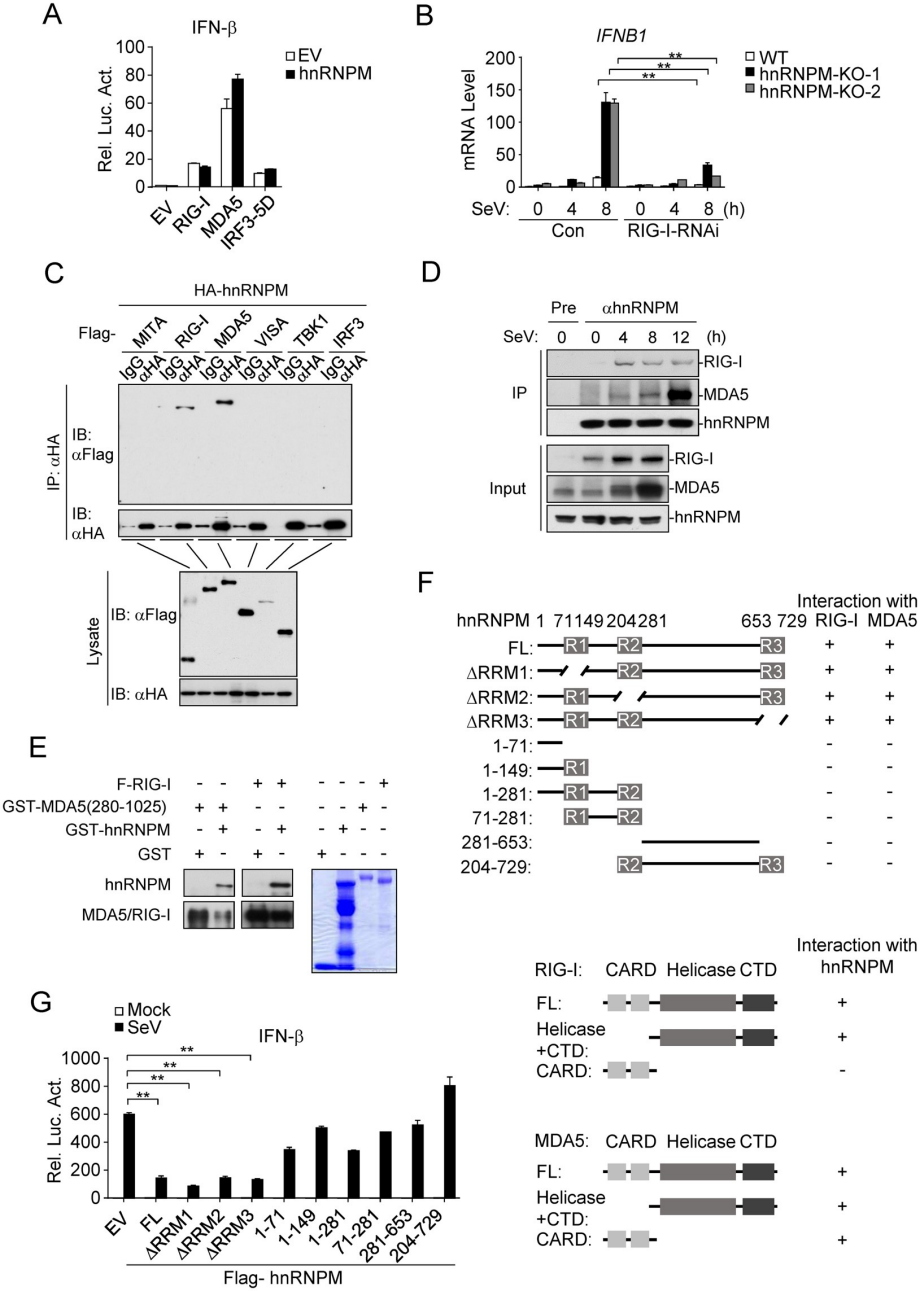
hnRNPM interacts with RLRs. (A) Effects of hnRNPM on activation of the *IFNB* promoter induced by overexpression of various components. HEK293 cells were co-transfected with the indicated plasmids with empty vector (EV) or Flag-tagged hnRNPM plasmids for 20 h before luciferase assays. (B) Effects of RIG-I knockdown on SeV-induced transcription of *IFNB1* gene in hnRNPM-KO HEK293 cells. hnRNPM-KO and control HEK293 cells were transfected with RIG-I-shRNA plasmid and selected with puromycin (1 μg/ml) for 2 days and then infected with SeV for the indicated times before qPCR analysis. (C) hnRNPM interacts with RIG-I and MDA5. HEK293 cells were transfected with the indicated plasmids before co-immunoprecipitation and immunoblotting analysis. (D) Endogenous hnRNPM interacts with RIG-I and MDA5. HEK293 cells were treated with IFN-β (50ng/ml) for 1 hour and then left un-infected or infected SeV for indicated times before co-immunoprecipitation and immunoblotting analysis. (E) hnRNPM interacts with RIG-I and MDA 5 in *vitro*. The indicated recombinant protein were incubated in PBS with RNase I. Lysates were respectively immunoprecipitated with anti-MDA5 or anti-Flag affinity gel. Bound proteins were analyzed by immunoblots with the indicated antibodies. GST was used as negative control. The purified proteins were stained by Coomassie blue. (F) Domain mapping of the interactions between hnRNPM and RIG-I or MDA5. HEK293 cells were transfected with the indicated plasmids before co-immunoprecipitation and immunoblotting analysis with the indicated antibodies. The results were schematically presented. The blots were shown in S1 Fig. FL, full length. (G) Effects of hnRNPM mutants on SeV-induced activation of the *IFNB* promoter. HEK293 cells were transfected with the indicated hnRNPM mutant plasmids and then infected with SeV for 12 h before luciferase assays. **p < 0.01 (unpaired t test).

We next investigated the molecular mechanisms that are responsible for the roles of hnRNPM in innate immune response to RNA virus. Results of reporter assays showed that overexpression of hnRNPM had no marked effects on RIG-I- and MDA5-mediated activation of the IFN-β promoter (Fig 5A). Furthermore, knockdown of RIG-I inhibited the induction of *IFNB* mRNA by SeV in hnRNPM-deficient cells (Fig 5B). In transient transfection and co-immunoprecipitation experiments, hnRNPM interacted with RIG-I and MDA5, but not with MITA, VISA, TBK1 and IRF3 (Fig 5C). Overexpression of hnRNPM had no effects on interaction between RIG or MDA5 with VISA (S2A Fig). Endogenous co-immunoprecipitation experiments indicated that hnRNPM interacted with RIG-I/MDA5 in a viral-infection-dependent manner (Fig 5D). We have further produced recombinant hnRNPM, RIG-I and MDA5 (280–1,025). *Vitro* protein pull-down analysis showed that hnRNPM can directly interact with RIG-I or MDA5 in RNA-free condition (Fig 5E). These results suggest that hnRNPM acts at the level of RLRs.

Both RIG-I and MDA5 contain two N-terminal CARD domains, a middle helicase domain, and a CTD, whereas hnRNPM contains three RNA recognition motif (RRM) domains (Fig 5F). Domain mapping experiments indicated that the CARD and the helicase-CTD of MDA5 could independently interact with hnRNPM, while the helicase-CTD but not CARD of RIG-I was responsible for its interaction with hnRNPM (Fig 5F and S2B Fig). Furthermore, we found that deletion of an individual RRM domain of hnRNPM had no marked effects on its interaction with RIG-I or MDA5. However, all other examined deletion mutants of hnRNPM failed to interact with RIG-I or MDA5 (Fig 5F and S2C Fig). Reporter assays showed that wild-type hnRNPM and its mutants that interacted with RIG-I or MDA5 but not the other mutants inhibited SeV-induced activation of the IFN-β promoter (Fig 5G). These results suggest that the association of hnRNPM with RIG-I or MDA5 mediates its inhibition of RLR-mediated signaling.

### hnRNPM binds synthetic and viral RNA

**Fig 6:**
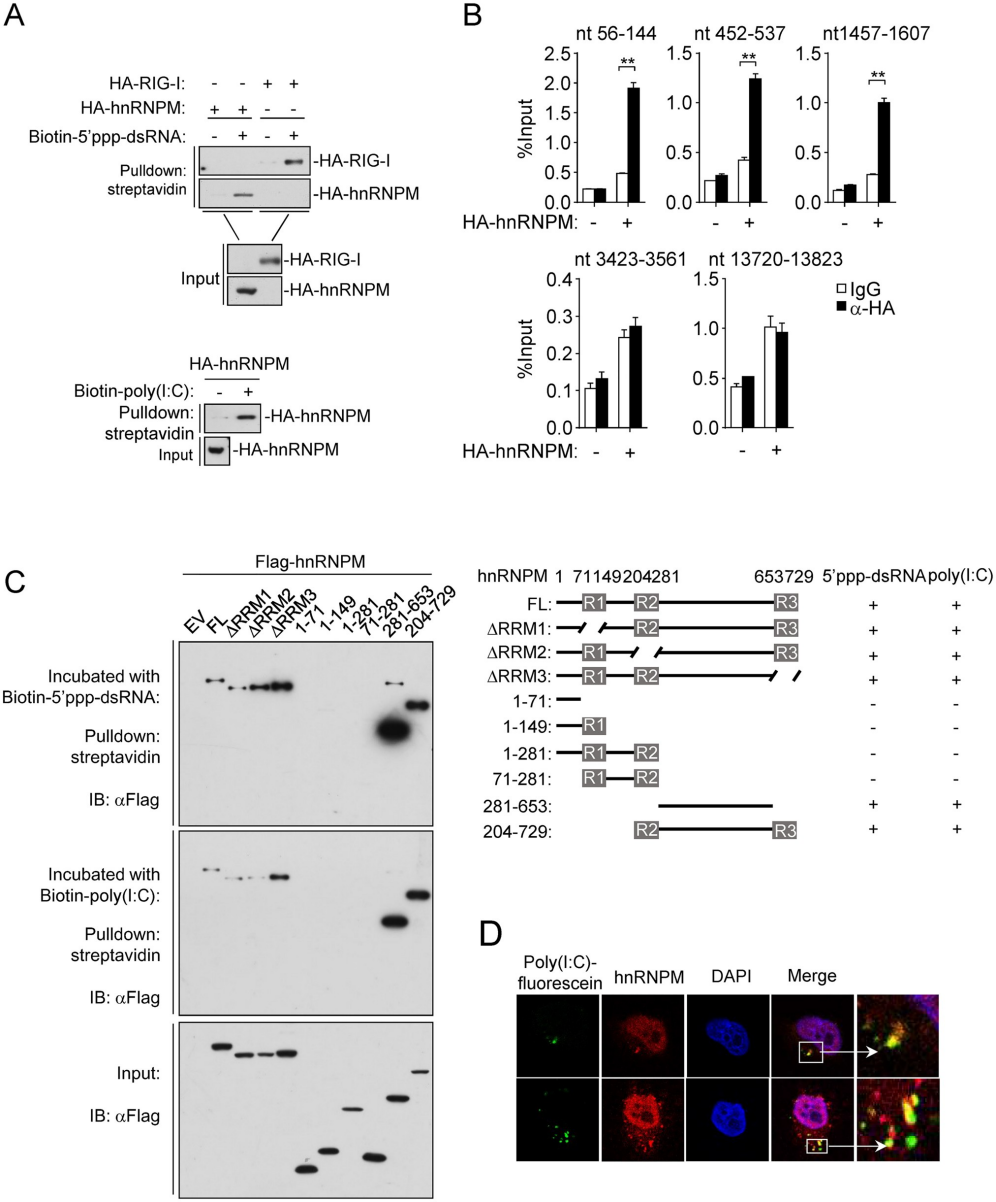
hnRNPM recognizes synthetic or viral RNA. (A) hnRNPM binds to 5’ppp-dsRNA and poly(I:C). HEK293 cells were transfected with the indicated plasmids. Twenty hours later, the cell lysates were incubated with the indicated biotinylated nucleic acids and streptavidin-sepharose beads for *in vitro* pull-down assays. The bound proteins were then analyzed by immunoblots with anti-HA. (B) hnRNPM binds to SeV RNA. HEK293 cells (2x10^6^) were transfected with the indicated plasmids. At 20 h after transfection, cells were infected with SeV for 3 h. Cell lysates were then immunoprecipitated with control IgG or anti-HA. The immunoprecipitates were treated with RNase I and bound-RNA was extracted for qPCR analysis. Results with addition primers targeting SeV RNA were shown in S2 Fig. **p < 0.01 (unpaired t test). nt, nucleotides. (C) The binding of hnRNPM and its truncations with 5’ppp-dsRNA and poly(I:C). HEK293 cells (2x10^6^) were transfected with the indicated plasmids. Cell lysates were incubated with biotinylated-5’ppp-dsRNA or biotinylated-poly(I:C) and streptavidin-sepharose. Bound proteins were analyzed by immunoblots with the indicated antibodies. (D) Confocal microcopy analysis of colocalization of poly(I:C) and hnRNPM. HeLa cells were transfected with poly(I:C)-fluorescein for 6h and then immunostaining of hnRNPM (red). Scale bars represent 50 μm.

Since hnRNPM is a heterogeneous nuclear ribonucleoprotein that contains three RRMs [38], we determined whether hnRNPM binds to viral RNA similarly as RIG-I and MDA5. Previously, it has been shown that the CTD of RIG-I binds to 5’ppp-ssRNA, 5’ppp-dsRNA, and short blunt-ended dsRNA, with significantly higher affinity for 5’ppp-dsRNA [39], whereas the CTD of MDA5 has higher affinity to long dsRNA such as synthetic poly(I:C). Pull-down experiments indicated that ectopically-expressed hnRNPM could bind to 5’ppp-dsRNA and poly(I:C) (Fig 6A). We also examined whether hnRNPM binds to viral RNA in infected cells by ‘‘footprint” experiments [30], [40]. After SeV infection (which is recognized by both RIG-I and MDA5), we immunoprecipitated hnRNPM and the immunoprecipitates were treated with RNase I. The protein-protected viral RNA was detected by RT-PCR with primers targeting various regions of SeV RNA. The results showed that hnRNPM could bind to naturally infected viral RNA similar as RIG-I and MDA5 (Fig 6B, S3 & S4 Figs). Interestingly, hnRNPM appeared preferly to bind to the 5’- terminus of SeV RNA (Fig 6B, S3 & S4 Figs) and had a higher affinity with viral RNA in the late phase of infection (S4 Fig). Confocal microscopy analysis confirmed the colocation between poly(I:C) and hnRNPM (Fig 6D). These experiments suggest that hnRNPM can directly bind to synthetic and viral RNA. Furthermore, pull-down experiments revealed that the individual RRM-deleted mutants of hnRNPM as well as hnRNPM (aa281-653) and hnRNPM (aa204-729) could bind to 5’ppp-dsRNA or SeV RNA (Fig 6C). These results indicate that RRM regions that bind cellular and viral RNA are different. Notably, although hnRNPM (aa281-653) and hnRNPM (aa204-729) could bind to viral RNA, they lost the ability to inhibit SeV-induced activation of the IFN-β promoter (Fig 5G). These findings suggest that the binding of hnRNPM to viral RNA is insufficient for regulating RLR-mediated signaling.

### hnRNPM inhibits detection of viral RNA by RIG-I and MDA5

**Fig 7:**
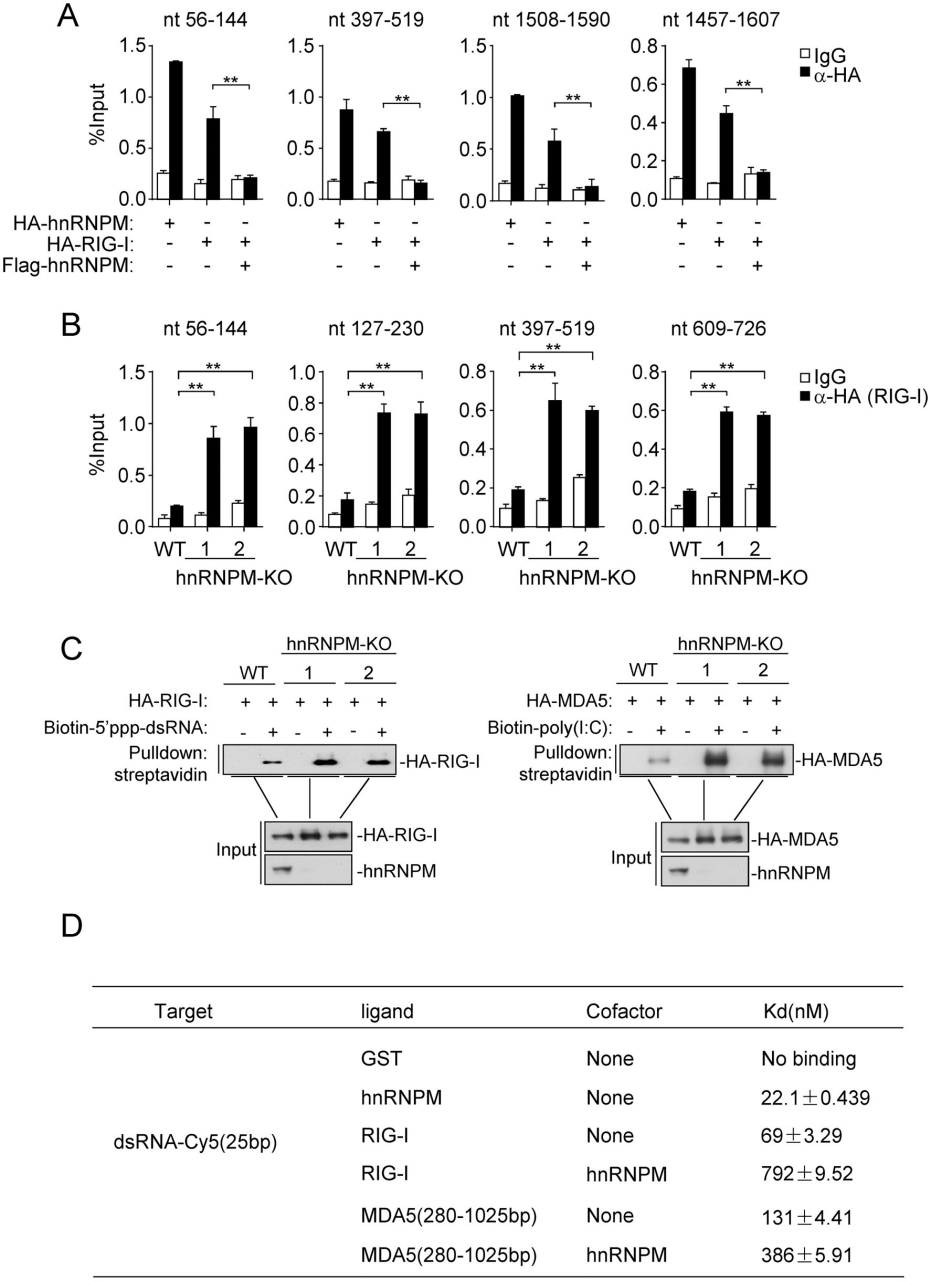
hnRNPM inhibits sensing of viral RNA by RIG-I and MDA5. (A) hnRNPM inhibits the binding of RIG-I to SeV RNA. HEK293 cells (2x10^6^) were transfected with the indicated plasmids. Twenty hours after transfection, cells were infected with SeV for 3 h. Cell lysates were then immunoprecipitated with control IgG or anti-HA. The immunoprecipitates were treated with RNase I and bound-RNA was extracted for qPCR analysis. Results with other examined primers targeting SeV RNA were shown in S5A Fig. nt, nucleotides. (B) hnRNPM-deficiency facilitates the binding of RIG-I to SeV RNA. hnRNPM-KO and control HEK293 cells (1x10^7^) were transfected with HA-tagged RIG-I. Twenty hours after transfection, cells were infected with SeV for 3 h. Cell lysates were collected for RIP assays similarly as in (A). Results with other examined primers targeting SeV RNA were shown in S3B Fig. (C) hnRNPM-deficiency facilitates the binding of RIG-I and MDA5 to 5’ppp-dsRNA or poly(I:C) respectively. hnRNPM-KO and control HEK293 cells (1x10^7^) were transfected with HA-tagged RIG-I or MDA5 (10 μg each). Twenty hours after transfection, cells were collected for RNA pull-down assays similarly as in Fig 6(A). (D) Binding affinities of recombinant hnRNPM, RIG-I and MDA5(280–1025) to dsRNA. The affinities were measured by MST as described in the Methods. **p < 0.01 (unpaired t test).

Finally, we investigated whether hnRNPM regulates sensing of viral RNA by RIG-I and MDA5. The ‘‘footprint” experiments showed that overexpression of hnRNPM inhibited the binding of RIG-I to SeV RNA (Fig 7A and S5A Fig). Conversely, deficiency of hnRNPM enhanced the binding of RIG-I to SeV RNA (Fig 7B and S5B Fig). Furthermore, pull-down experiments also indicated that the binding of RIG-I to 5’ppp-dsRNA or MDA5 to poly(I:C) was enhanced in hnRNPM-deficient cells in comparison to control cells (Fig 7C). We further produced recombinant hnRNPM and MDA5(280–1,025) in bacteria and immunoprecipitated Flag-tagged RIG-I from transfected HEK 293 cells for microscale thermophoresis technology (MST) experiments in vitro. The results showed that hnRNPM bound to dsRNA (25 bp) with an affinity of Kd = 22.1 ± 0.439 nM, which was higher than that of MDA5 (Kd = 131 ± 4.41 nM) and RIG-I (Kd = 69 ± 3.29 nM) with dsRNA (Fig 7D and S5C Fig). Interestingly, recombinant hnRNPM caused approximately 10- and 3-fold decrease of the affinities of RIG-I and MDA5 to dsRNA respectively (Fig 7D and S5C Fig). These results suggest that hnRNPM impairs the binding of RIG-I and MDA5 to viral RNA.

## Discussion

Recognition of viral RNA by RLRs is essential for the initiation of innate antiviral response initiation. Although the regulation of RLR-mediated downstream signaling have been extensively investigated, little is known about the regulatory mechanisms of the recognition of viral RNA by RLRs. Recently, we identified ZCCHC3 as a co-receptor for RIG-I and MDA5 by facilitating the sensing of viral RNA [30]. In addition, MEX3C and RAVER1 have been reported to facilitate the recognition of viral RNA by RIG-I and MDA5 respectively [28, 29]. However, how these processes are negatively regulated remains enigmatic. In the current study, we identified hnRNPM as an inhibitor of RLR-mediated innate immune response by impairing the binding of RIG-I and MDA5 to viral RNA.

Overexpression of hnRNPM inhibited SeV-triggered activation of ISRE, NF-κB, and the IFN-β promoter, while deficiency of hnRNPM had the opposite effects. In addition, the replication of RNA virus was decreased in hnRNPM-deficient cells compared with control cells. These data established a critical role for hnRNPM in innate immune response to viral RNA.

We found that hnRNPM underwent re-distribution between nucleus and cytoplasm during RNA virus infection. hnRNPM was mostly localized in the nucleus in rest cells. Following infection, hnRNPM was translocated from the nucleus to cytoplasm, which was impaired in RIG-I knockdown cells. Furthermore, hnRNPM was also translocated from the nucleus to cytoplasm in IFN-β-treated cells. These results suggest that hnRNPM translocation is dependent on RLRs.

Several evidences suggest that hnRNPM inhibits RNA virus-triggered innate immunity by antagonizing RNA sensing of RLRs. Firstly, viral infection caused export of hnRNPM from the nucleus to cytoplasm, indicating a cytoplasmic role of hnRNPM after viral infection. Second, hnRNPM was associated with RIG-I and MDA5 and their interactions were important for the functions of hnRNPM. Third, overexpression of hnRNPM inhibited the binding of RIG-I to SeV RNA. Conversely, deficiency of hnRNPM enhanced the binding of RIG-I to SeV RNA. Recombinant hnRNPM caused approximately 10- and 3-fold decrease of the affinities of RIG-I and MDA5 to dsRNA respectively. These results collectively suggest that hnRNPM is an inhibitor for RLR-mediated innate immune response by impairing viral RNA sensing by RIG-I and MDA5.

Our results revealed that the classical cellular mRNA binding regions (RRM) of hnRNPM differs from its viral RNA binding regions. The mutants containing partial RRM regions such as 1–149, 1–281 and 71–281 are lack of viral RNA binding activity while the mutant 281–653 being lack of all three RRM domains had a stronger affinity to viral RNA.

Although hnRNPM binds to dsRNA, the binding is insufficient for regulating RLR-mediated signaling. Several truncations of hnRNPM, aa281-653 and aa204-729, that could bind to RNA but failed to interact with RIG-I and MDA5, had no effects on SeV-triggered activation of the IFNB promoter. hnRNPM appeared to prefer to bind to the 5’- terminus of SeV RNA, but inhibited the binding of RIG-I to diverse regions of SeV RNA. The regions of RIG-I that interact with hnRNPM overlapped with its viral RNA binding regions. These results collectively suggest the functions of hnRNPM on RLR-mediated innate immune response are mostly dependent on its association with RLRs instead of RNA binding.

Interestingly, we also found that hnRNPM deficiency enhanced DNA virus -triggered expression of downstream genes. However, hnRNPM deficiency had no significant effects on the induction of downstream genes by transfected DNA mimics such as HSV120, HT-DNA and VACA70. These results suggest that hnRNPM regulates HSV-1-triggered innate immune responses in a viral DNA-independent manner. Previous studies have implicated that RIG-I also detected dsRNA produced by herpesviruses [41–45]. We propose a possibility that hnRNPM is partly involved in HSV-1-triggered innate immune signaling by regulating the sensing of HSV-1-derived RNA by RIG-I.

In conclusion, our study suggests that hnRNPM is a decoy of innate antiviral response by impairing the sensing of viral RNA by RLRs, which provides a critical control mechanism of viral RNA sensing for the host to avoid excessive immune response.

## Materials and methods

### Reagents, antibodies, cells and viruses

Poly(dA:dT), poly(I:C)-HWM, poly(I:C)-LWM, poly(I:C)-fluorescein, 5’ppp-dsRNA, RNase inhibitor, M-MLV and Lipofectamine 2000 (Invivogen); HT-DNA (Sigma); DTT (Fermentas); RNase I (Ambion); polybrene (Millipore); recombinant IFN-β (PeproTech); SYBR (Bio-Rad); dual-specific luciferase assay kit (Promega); puromycin and EZ-link psoralen-PEG_3_-biotin and streptavidin agarose resin (Thermo); protein G sepharose (GE Healthcare); anti-Flag affinity gel (Biomake, B73102); RNAiso plus (Takara); and recombinant IFN-β (R&D systems) were purchased from the indicated companies.

Anti-Flag (F3165), anti-β-actin (A2228) and anti-β-tubulin (T8328) were from Sigma-Aldrich. Anti-phospho-IκBα (Ser32/36) (5A5), anti-phospho-IRF3 (Ser396) (4D4G), anti-phospho-STAT1 (Tyr701) (58D6) and anti-phospho-p65 (Ser536) were from Cell Signaling Technology. Anti-LMNB1 (12987-1-AP) was from ProteinTech. Anti-G3BP1(210-323aa) (611126) was from BD Biosciences. Anti-HA (16B12) was from Covance. Anti-TBK1 (ab109735), anti-phospho-TBK1 (Ser172) (ab109272) were from Abcam. Anti-IRF3 (FL-425), anti-p65 (C-20) (sc-372), anti-STAT1 (C-111) were from Santa Cruz Biotechnology. Goat anti-mouse IgG (R37116) or donkey anti–rabbit (R37119) conjugated to Alexa Fluor 594 and goat anti-mouse IgG (A-10684) conjugated to Alexa Fluor 488 were purchased from Thermo Fisher. Mouse antisera against hnRNPM, IκBα, RIG-I and MDA5 were raised against purified recombinant human hnRNPM (1-729aa), IκBα, RIG-I (1-200aa) and MDA5 (1-200aa). Rabbit antisera against SeV was raised against purified SeV.

Human embryonic kidney 293 (HEK293, CRL-1573), Human acute monocytic leukemia cell line (THP-1) and Henrietta Lacks (HeLa, CCL-2) cells were purchased from American Type Culture Collection, and Vero cells were purchased from China Center for Type Culture Collection (Wuhan, China). HEK293T cells were originally provided by G. Johnson (National Jewish Health, Denver, CO). The strains (BL21 and DH5α) were purchased from ATCC.

SeV, VSV (Indiana Strain), NDV and HSV-1 were previously described [46, 47]. EMCV was provided by Dr. H. Yang (China Agricultural University).

### DNA oligonucleotides

The following oligonucleotides were used to stimulate cells:

VACV70: 5’-CCATCAGAAAGAGGTTTAATATTTTTGTGAGACCATGGA

AGAGAGAAAGAGATAAAACTTTTTTACGACT-3’;

HSV120: 5’-AGACGGTATATTTTTGCGTTATCACTGTCCCGGATTGGAC

ACGGTCTTGTGGGATAGGCATGCCCAGAAGGCATATTGGGTTAACCCCT

TTTTATTTGTGGCGGGTTTTTTGGAGGACTT-3’.

### Constructs

RIG-I or MDA5 and their mutants, Flag or HA-tagged hnRNPM and their truncation mutants, pGEX6p-1-GST-hnRNPM and pGEX6p-1-GST-MDA5 (280–1025) were constructed by standard molecular biology techniques. The other expression and reporter plasmids were previously described [9].

### Transfection and reporter assays

HEK293 cells were transfected by standard calcium phosphate precipitation method. To normalize for transfection efficiency, 0.01 μg of pRL-TK (Renilla luciferase) reporter plasmid was added to each transfection. Luciferase assays were performed using a dual-specific luciferase assay kit. Firefly luciferase activities were normalized on the basis of Renilla luciferase activities.

### RNA interference

Double-stranded oligonucleotides corresponding to the target sequences were cloned into the pSuper. Retro-RNAi plasmid (Oligoengine).

The following sequences were targeted for hnRNPM mRNA.

The hnRNPM-shRNA#1 targeting sequence: 5’-GGCATAGGATTTGGAATAA-3’

The hnRNPM-shRNA#2 targeting sequence: 5’-GCAATCGCTTTGAGCCATA-3’.

The RIG-I-shRNA targeting sequence: 5’-CCGTGATTCCACTTTCCTG-3’

### Generation of hnRNPM CRISPR knockout cells

HEK293 or THP-1 cells were transduced with plentiCRISPRv2-hnRNPM-sgRNA viruses for five days. Puromycin-resistant individual clones were selected and analyzed by immunoblotting to determine the efficiency of hnRNPM knockout.

The hnRNPM-gRNA#1 targeting sequence: 5’-GGCGACGGAGATCAAAATGG-3’.

The hnRNPM-gRNA#2 targeting sequence: 5’-GGCGGCGACGGAGATCAAAA-3’.

### qPCR

Total RNA was isolated for qPCR analysis to measure mRNA abundance of the indicated genes. Data shown are the relative abundance of the indicated mRNA derived from human cells normalized to that GAPDH respectively. Gene-specific primer sequences were as described [46, 47].

Q-PCR primers for *HNRNPM*:

Forward Sequence: TCCTGAACGCCCACAGCAACTT

Reverse Sequence: TGCCTTTGCTCAGATGGTTGGC

### Co-immunoprecipitation and immunoblot analysis

Cells were lysed in NP-40 lysis buffer (20 mM Tris-HCl (pH 7.4), 150 mM NaCl, 1mM EDTA, 1% NonidetP-40, 10 μg/ml aprotinin, 10 μg/ml leupeptin, and 1 mM phenyl-methylsulfonyl fluoride). The lysates were subjected to immunoprecipitation and immunoblotting analysis with the indicated antibodies.

### Subcellular fractionation

Nuclear and cytoplasmic fractions extraction of HEK293 cells were generated according to the instruction of Nuclear and Cytoplasmic Protein Extraction Kit (PPLYGEN, P1201).

### In *vitro* RNA pull-down assays

Poly(I:C) and 5’ppp-dsRNA was conjugated to biotin by UV (365 nm wave-length) cross-linking for 1 hour. HEK293 cells transfected with the indicated plasmids were lysed in Pre-lysis buffer pre-treated with DEPC. Lysates were incubated with biotinylated-5’ppp-dsRNA or biotinylated-poly(I:C) for 2 hours at room temperature, and then incubated with streptavidin beads for another 1 hour at room temperature. The beads were washed four times with lysis buffer and analyzed by immunoblots with the indicated antibodies.

### Recombinant protein purification

The pGEX-6p-1-GST plasmids encoding hnRNPM and MDA5(280–1025) were transformed into BL21 competent cells. Expression of the proteins was induced with 0.1 mM IPTG at 16°C for 24 hours. The proteins were purified with GST resins and eluted with elution buffer (PBS, 100 mM Tris-HCl pH 8.8, 40mM reduced glutathione). To obtain purified RIG-I, Flag-RIG-I plasmid was transfected into HEK293 cells by standard calcium phosphate precipitation method. The expressed Flag-RIG-I protein was immunoprecipitated with anti-Flag affinity gel and eluted with 3×Flag peptides.

### In *vitro* protein pull-down assays

Purified recombinant protein hnRNPM and MDA5/RIG-I were incubated in PBS with RNase I at 4°C for 3 hours. Lysates were respectively immunoprecipitated with anti-MDA5 and protein G beads (50 ul) or anti-Flag affinity gel at 4°C for another 2 hours. GST was used as negative control. Bound proteins were analyzed by immunoblots with the indicated antibodies.

### Microscale thermophoresis technology (MST)

MST analysis was performed using a NanoTemper Monolith NT.115 instrument (NanoTemper Technologies GmbH). For detecting affinity between dsRNA and GST-hnRNPM, Flag-RIG-I or GST-MDA5, 20 nM Cy5-labeled 25 bp dsRNA (Sangon Biotech, China) was mixed with different concentrations of proteins in PBS with 100 mM Tris-HCl (pH 8.8). GST was used as negative control. Samples were loaded into Premium Coated Capillaries and MST measurements were performed using 20% MST power and 40% LED power at 25°C. Laser-on and -off times were 30 and 5 s respectively. NanoTemper Analysis 1.2.20 software was used to fit the data and to determine the apparent Kd values.

### RNA-binding protein immunoprecipitation (RIP)

HEK293 cells were transfected with the indicated HA- or Flag-tagged plasmids for 20 hours, then infected with SeV for 1 hour, washed with medium and cultured for 2 more hours. Cell lysates were immunoprecipitated with IgG or anti-HA (2 μg) and protein G beads (50 μl) at 4°C for 3 hours. The immunoprecipitates were treated with diluted RNase I (1:25 in PBS) at 37°C for 5 min. The bead-bound immunoprecipitates were washed for 3 times with lysis buffer containing RNase inhibitors. The protein and RNA complexes were eluted with 200 μL TE buffer containing 10 mM DTT at 37°C for 30 min. The RNA was extracted using Trizol reagent before qPCR analysis for SeV RNA. The SeV genome primer sequences were described in S1 Table.

### Confocal microscopy

Confocal microscopy was performed as previously described [48]. Briefly, cells infected with virus or transfected poly(I:C) for the indicated times were fixed with 4% paraformaldehyde for 10 min at 25°C and then permeabilized and stained with indicated antibodies by standard protocols. The stained cells were observed with a ZEISS confocal microscope under a 100× oil objective.

### Viral plaque assay

Host cells (5×10^5^) cultured in 12-well plates were infected with viruses at the respective MOI for 1 hour, then washed with PBS and cultured with 1 ml fresh complete medium. The plates were incubated for 36 hours post-infection at 37°C, 10% CO_2_. The media were collected and used for plaque assays on monolayers of Vero cells seeded in 24-well plates. The Vero cells were infected by incubation for 1 hour at 37°C with serial dilutions of collected media. After infection for 1 hour, the cells were overlaid with 1.5% methylcellulose and then incubated for about 48 hours. The overlay was removed, and cells were fixed with 4% paraformaldehyde for 15 min and stained with 1% crystal violet for 30 min before plaque counting.

### Statistical analysis

Unpaired Student’s t test was used for statistical analysis with GraphPad Prism Software. p < 0.05 was considered significant.

## Supporting information

S1 Fig Effects of hnRNPM-deficiency on DNA-induced innate immunity. (A) Effects of hnRNPM-deficiency on HSV-1-induced transcriptions of downstream genes. hnRNPM-KO and control THP-1 cells were left un-infected or infected with HSV-1 for the indicated times before qPCR analysis. (B) Effects of hnRNPM-deficiency on cytosolic dsDNA-induced transcriptions of downstream genes. hnRNPM-KO and control THP-1 cells were transfected with the indicated nucleic acids (2 μg/ml) for 4 h before qPCR analysis.
(TIF)

S2 Fig Interaction between hnRNPM and RIG-I or MDA5. (A) hnRNPM had no effect on the interaction of RLR with their adaptor. HEK293 cells were transfected with the indicated plasmids before co-immunoprecipitation and immunoblotting analysis with the indicated antibodies. (B&C) Domain mapping of the interactions between hnRNPM and RIG-I or MDA5.HEK293 cells were transfected with the indicated plasmids before co-immunoprecipitation and immunoblotting analysis with the indicated antibodies. The results were schematically presented in Fig 5D. FL, full length.
(TIF)

S3 Fig hnRNPM binds to SeV RNA. Supplementary data for Fig 6B. **p < 0.01 (unpaired t test).
(TIF)

S4 Fig Endogenous hnRNPM binds to SeV RNA. HEK293 cells were infected with SeV for indicated times. Cell lysates were then immunoprecipitated with control IgG or anti-hnRNPM. The immunoprecipitates were treated with RNase I and bound-RNA was extracted for qPCR analysis. nt, nucleotides.
(TIF)

S5 Fig hnRNPM inhibits sensing of viral RNA by RIG-I and MDA5. (A) Supplementary data for Fig 7A. (B) Supplementary data for Fig 7B. (C) Supplementary data to Fig 7D. *p < 0.05, **p < 0.01 (unpaired t test).
(TIF)

S1 Table The Q-PCR primers for SeV genome. The SeV genome primer sequences used in Q-PCR were described in the table.
(DOC)

## References

[1] Takeuchi O, Akira S. Pattern recognition receptors and inflammation. Cell. 2010;140(6):805–20. Epub 2010/03/23. 10.1016/j.cell.2010.01.022 . 20303872

[2] Loo YM, Gale M Jr. Immune signaling by RIG-I-like receptors. Immunity. 2011;34(5):680–92. Epub 2011/05/28. 10.1016/j.immuni.2011.05.003 . 21616437 PMC3177755

[3] Lester SN, Li K. Toll-like receptors in antiviral innate immunity. J Mol Biol. 2014;426(6):1246–64. Epub 2013/12/10. 10.1016/j.jmb.2013.11.024 . 24316048 PMC3943763

[4] Liu Y, Olagnier D, Lin R. Host and Viral Modulation of RIG-I-Mediated Antiviral Immunity. Front Immunol. 2016;7:662 Epub 2017/01/18. 10.3389/fimmu.2016.00662 . 28096803 PMC5206486

[5] Yoneyama M, Fujita T. RNA recognition and signal transduction by RIG-I-like receptors. Immunol Rev. 2009;227(1):54–65. Epub 2009/01/06. 10.1111/j.1600-065X.2008.00727.x . 19120475

[6] Kawai T, Takahashi K, Sato S, Coban C, Kumar H, Kato H, et al IPS-1, an adaptor triggering RIG-I- and Mda5-mediated type I interferon induction. Nat Immunol. 2005;6(10):981–8. Epub 2005/08/30. 10.1038/ni1243 . 16127453

[7] Meylan E, Curran J, Hofmann K, Moradpour D, Binder M, Bartenschlager R, et al Cardif is an adaptor protein in the RIG-I antiviral pathway and is targeted by hepatitis C virus. Nature. 2005;437(7062):1167–72. Epub 2005/09/24. 10.1038/nature04193 . 16177806

[8] Seth RB, Sun L, Ea CK, Chen ZJ. Identification and characterization of MAVS, a mitochondrial antiviral signaling protein that activates NF-kappaB and IRF 3. Cell. 2005;122(5):669–82. Epub 2005/08/30. 10.1016/j.cell.2005.08.012 . 16125763

[9] Xu LG, Wang YY, Han KJ, Li LY, Zhai Z, Shu HB. VISA is an adapter protein required for virus-triggered IFN-beta signaling. Mol Cell. 2005;19(6):727–40. Epub 2005/09/13. 10.1016/j.molcel.2005.08.014 . 16153868

[10] Hou F, Sun L, Zheng H, Skaug B, Jiang QX, Chen ZJ. MAVS forms functional prion-like aggregates to activate and propagate antiviral innate immune response. Cell. 2011;146(3):448–61. Epub 2011/07/26. 10.1016/j.cell.2011.06.041 . 21782231 PMC3179916

[11] Liu S, Chen J, Cai X, Wu J, Chen X, Wu YT, et al MAVS recruits multiple ubiquitin E3 ligases to activate antiviral signaling cascades. Elife. 2013;2:e00785 Epub 2013/08/21. 10.7554/eLife.00785 . 23951545 PMC3743401

[12] Hornung V, Ellegast J, Kim S, Brzozka K, Jung A, Kato H, et al 5'-Triphosphate RNA is the ligand for RIG-I. Science. 2006;314(5801):994–7. Epub 2006/10/14. 10.1126/science.1132505 . 17038590

[13] Kato H, Takeuchi O, Mikamo-Satoh E, Hirai R, Kawai T, Matsushita K, et al Length-dependent recognition of double-stranded ribonucleic acids by retinoic acid-inducible gene-I and melanoma differentiation-associated gene 5. J Exp Med. 2008;205(7):1601–10. Epub 2008/07/02. 10.1084/jem.20080091 . 18591409 PMC2442638

[14] Pichlmair A, Schulz O, Tan CP, Naslund TI, Liljestrom P, Weber F, et al RIG-I-Mediated Antiviral Responses to Single-Stranded RNA Bearing 5'-Phosphates. Science. 2006;314(5801):997–1001. 10.1126/science.1132998 17038589

[15] Kato H, Takeuchi O, Sato S, Yoneyama M, Yamamoto M, Matsui K, et al Differential roles of MDA5 and RIG-I helicases in the recognition of RNA viruses. Nature. 2006;441(7089):101–5. Epub 2006/04/21. 10.1038/nature04734 . 16625202

[16] Loo YM, Fornek J, Crochet N, Bajwa G, Perwitasari O, Martinez-Sobrido L, et al Distinct RIG-I and MDA5 signaling by RNA viruses in innate immunity. J Virol. 2008;82(1):335–45. Epub 2007/10/19. 10.1128/JVI.01080-07 . 17942531 PMC2224404

[17] Cui J, Song Y, Li Y, Zhu Q, Tan P, Qin Y, et al USP3 inhibits type I interferon signaling by deubiquitinating RIG-I-like receptors. Cell Res. 2014;24(4):400–16. Epub 2013/12/25. 10.1038/cr.2013.170 . 24366338 PMC3975496

[18] Fan Y, Mao R, Yu Y, Liu S, Shi Z, Cheng J, et al USP21 negatively regulates antiviral response by acting as a RIG-I deubiquitinase. J Exp Med. 2014;211(2):313–28. Epub 2014/02/05. 10.1084/jem.20122844 . 24493797 PMC3920558

[19] Gack MU, Shin YC, Joo CH, Urano T, Liang C, Sun L, et al TRIM25 RING-finger E3 ubiquitin ligase is essential for RIG-I-mediated antiviral activity. Nature. 2007;446(7138):916–20. Epub 2007/03/30. 10.1038/nature05732 . 17392790

[20] Hu MM, Liao CY, Yang Q, Xie XQ, Shu HB. Innate immunity to RNA virus is regulated by temporal and reversible sumoylation of RIG-I and MDA5. J Exp Med. 2017;214(4):973–89. Epub 2017/03/03. 10.1084/jem.20161015 . 28250012 PMC5379974

[21] Narayan K, Waggoner L, Pham ST, Hendricks GL, Waggoner SN, Conlon J, et al TRIM13 is a negative regulator of MDA5-mediated type I interferon production. J Virol. 2014;88(18):10748–57. Epub 2014/07/11. 10.1128/JVI.02593-13 . 25008915 PMC4178852

[22] Oshiumi H, Miyashita M, Matsumoto M, Seya T. A distinct role of Riplet-mediated K63-Linked polyubiquitination of the RIG-I repressor domain in human antiviral innate immune responses. PLoS Pathog. 2013;9(8):e1003533 Epub 2013/08/21. 10.1371/journal.ppat.1003533 . 23950712 PMC3738492

[23] Pauli EK, Chan YK, Davis ME, Gableske S, Wang MK, Feister KF, et al The ubiquitin-specific protease USP15 promotes RIG-I-mediated antiviral signaling by deubiquitylating TRIM25. Sci Signal. 2014;7(307):ra3 Epub 2014/01/09. 10.1126/scisignal.2004577 . 24399297 PMC4008495

[24] Sun Z, Ren H, Liu Y, Teeling JL, Gu J. Phosphorylation of RIG-I by casein kinase II inhibits its antiviral response. J Virol. 2011;85(2):1036–47. Epub 2010/11/12. 10.1128/JVI.01734-10 . 21068236 PMC3020001

[25] Wang L, Zhao W, Zhang M, Wang P, Zhao K, Zhao X, et al USP4 positively regulates RIG-I-mediated antiviral response through deubiquitination and stabilization of RIG-I. J Virol. 2013;87(8):4507–15. Epub 2013/02/08. 10.1128/JVI.00031-13 . 23388719 PMC3624380

[26] Wies E, Wang MK, Maharaj NP, Chen K, Zhou S, Finberg RW, et al Dephosphorylation of the RNA sensors RIG-I and MDA5 by the phosphatase PP1 is essential for innate immune signaling. Immunity. 2013;38(3):437–49. Epub 2013/03/19. 10.1016/j.immuni.2012.11.018 . 23499489 PMC3616631

[27] Yan J, Li Q, Mao AP, Hu MM, Shu HB. TRIM4 modulates type I interferon induction and cellular antiviral response by targeting RIG-I for K63-linked ubiquitination. J Mol Cell Biol. 2014;6(2):154–63. Epub 2014/04/24. 10.1093/jmcb/mju005 . 24755855

[28] Kuniyoshi K, Takeuchi O, Pandey S, Satoh T, Iwasaki H, Akira S, et al Pivotal role of RNA-binding E3 ubiquitin ligase MEX3C in RIG-I-mediated antiviral innate immunity. Proc Natl Acad Sci U S A. 2014;111(15):5646–51. Epub 2014/04/08. 10.1073/pnas.1401674111 . 24706898 PMC3992669

[29] Chen H, Li Y, Zhang J, Ran Y, Wei J, Yang Y, et al RAVER1 is a coactivator of MDA5-mediated cellular antiviral response. J Mol Cell Biol. 2013;5(2):111–9. Epub 2013/02/08. 10.1093/jmcb/mjt006 . 23390309

[30] Lian H, Zang R, Wei J, Ye W, Hu MM, Chen YD, et al The Zinc-Finger Protein ZCCHC3 Binds RNA and Facilitates Viral RNA Sensing and Activation of the RIG-I-like Receptors. Immunity. 2018;49(3):438–48 e5. Epub 2018/09/09. 10.1016/j.immuni.2018.08.014 . 30193849

[31] Martinez-Contreras R, Cloutier P, Shkreta L, Fisette JF, Revil T, Chabot B. hnRNP proteins and splicing control. Adv Exp Med Biol. 2007;623:123–47. Epub 2008/04/03. . 18380344 10.1007/978-0-387-77374-2_8

[32] Han SP, Tang YH, Smith R. Functional diversity of the hnRNPs: past, present and perspectives. Biochem J. 2010;430(3):379–92. Epub 2010/08/28. 10.1042/BJ20100396 . 20795951

[33] Thomas P, Forse RA, Bajenova O. Carcinoembryonic antigen (CEA) and its receptor hnRNP M are mediators of metastasis and the inflammatory response in the liver. Clin Exp Metastasis. 2011;28(8):923–32. Epub 2011/09/09. 10.1007/s10585-011-9419-3 . 21901530

[34] Bajenova O, Stolper E, Gapon S, Sundina N, Zimmer R, Thomas P. Surface expression of heterogeneous nuclear RNA binding protein M4 on Kupffer cell relates to its function as a carcinoembryonic antigen receptor. Experimental Cell Research. 2003;291(1):228–41. 10.1016/s0014-4827(03)00373-2 14597422

[35] Marko M, Leichter M, Patrinou-Georgoula M, Guialis A. hnRNP M interacts with PSF and p54(nrb) and co-localizes within defined nuclear structures. Exp Cell Res. 2010;316(3):390–400. Epub 2009/10/31. 10.1016/j.yexcr.2009.10.021 . 19874820

[36] Onomoto K, Yoneyama M, Fung G, Kato H, Fujita T. Antiviral innate immunity and stress granule responses. Trends Immunol. 2014;35(9):420–8. Epub 2014/08/26. 10.1016/j.it.2014.07.006 . 25153707 PMC7185371

[37] Tsai WC, Lloyd RE. Cytoplasmic RNA Granules and Viral Infection. Annu Rev Virol. 2014;1(1):147–70. Epub 2014/11/03. 10.1146/annurev-virology-031413-085505 . 26958719 PMC4867093

[38] <The human hnRNP M proteins identification of a methioninearginine-rich repeat motif in ribonucleoproteins.pdf>. 10.1093/nar/21.3.439 PMC309137 8441656

[39] Lu C, Xu H, Ranjith-Kumar CT, Brooks MT, Hou TY, Hu F, et al The structural basis of 5' triphosphate double-stranded RNA recognition by RIG-I C-terminal domain. Structure. 2010;18(8):1032–43. Epub 2010/07/20. 10.1016/j.str.2010.05.007 . 20637642 PMC2919622

[40] Takahasi K, Yoneyama M, Nishihori T, Hirai R, Kumeta H, Narita R, et al Nonself RNA-sensing mechanism of RIG-I helicase and activation of antiviral immune responses. Mol Cell. 2008;29(4):428–40. Epub 2008/02/05. 10.1016/j.molcel.2007.11.028 . 18242112

[41] da Silva LF, Jones C. Small non-coding RNAs encoded within the herpes simplex virus type 1 latency associated transcript (LAT) cooperate with the retinoic acid inducible gene I (RIG-I) to induce beta-interferon promoter activity and promote cell survival. Virus Res. 2013;175(2):101–9. Epub 2013/05/08. 10.1016/j.virusres.2013.04.005 . 23648811 PMC4074922

[42] Jacquemont B, Roizman B. RNA synthesis in cells infected with herpes simplex virus. X. Properties of viral symmetric transcripts and of double-stranded RNA prepared from them. J Virol. 1975;15(4):707–13. Epub 1975/04/01. . 163916 10.1128/jvi.15.4.707-713.1975 PMC354512

[43] Rasmussen SB, Jensen SB, Nielsen C, Quartin E, Kato H, Chen ZJ, et al Herpes simplex virus infection is sensed by both Toll-like receptors and retinoic acid-inducible gene- like receptors, which synergize to induce type I interferon production. J Gen Virol. 2009;90(Pt 1):74–8. Epub 2008/12/18. 10.1099/vir.0.005389-0 . 19088275 PMC2956989

[44] Weber F, Wagner V, Rasmussen SB, Hartmann R, Paludan SR. Double-stranded RNA is produced by positive-strand RNA viruses and DNA viruses but not in detectable amounts by negative-strand RNA viruses. J Virol. 2006;80(10):5059–64. Epub 2006/04/28. 10.1128/JVI.80.10.5059-5064.2006 . 16641297 PMC1472073

[45] Zhao J, Zeng Y, Xu S, Chen J, Shen G, Yu C, et al A Viral Deamidase Targets the Helicase Domain of RIG-I to Block RNA-Induced Activation. Cell Host Microbe. 2016;20(6):770–84. Epub 2016/11/22. 10.1016/j.chom.2016.10.011 . 27866900 PMC5159239

[46] Luo WW, Li S, Li C, Lian H, Yang Q, Zhong B, et al iRhom2 is essential for innate immunity to DNA viruses by mediating trafficking and stability of the adaptor STING. Nat Immunol. 2016;17(9):1057–66. 10.1038/ni.3510 . 27428826

[47] Luo WW, Li S, Li C, Zheng ZQ, Cao P, Tong Z, et al iRhom2 is essential for innate immunity to RNA virus by antagonizing ER- and mitochondria-associated degradation of VISA. PLoS Pathog. 2017;13(11):e1006693 Epub 2017/11/21. 10.1371/journal.ppat.1006693 . 29155878 PMC5722342

[48] Luo WW, Lian H, Zhong B, Shu HB, Li S. Kruppel-like factor 4 negatively regulates cellular antiviral immune response. Cellular & molecular immunology. 2016;13(1):65–72. 10.1038/cmi.2014.125 . 25531393 PMC4711676

