# Supplementary figures and images for: The heterogeneous nuclear ribonucleoprotein hnRNPM inhibits RNA virus-triggered innate immunity by antagonizing RNA sensing of RIG-I-like receptors
Source: PLoS Pathog. 2019 Aug 21;15(8):e1007983. doi: 10.1371/journal.ppat.1007983 (PMC6703689; doi:10.1371/journal.ppat.1007983)

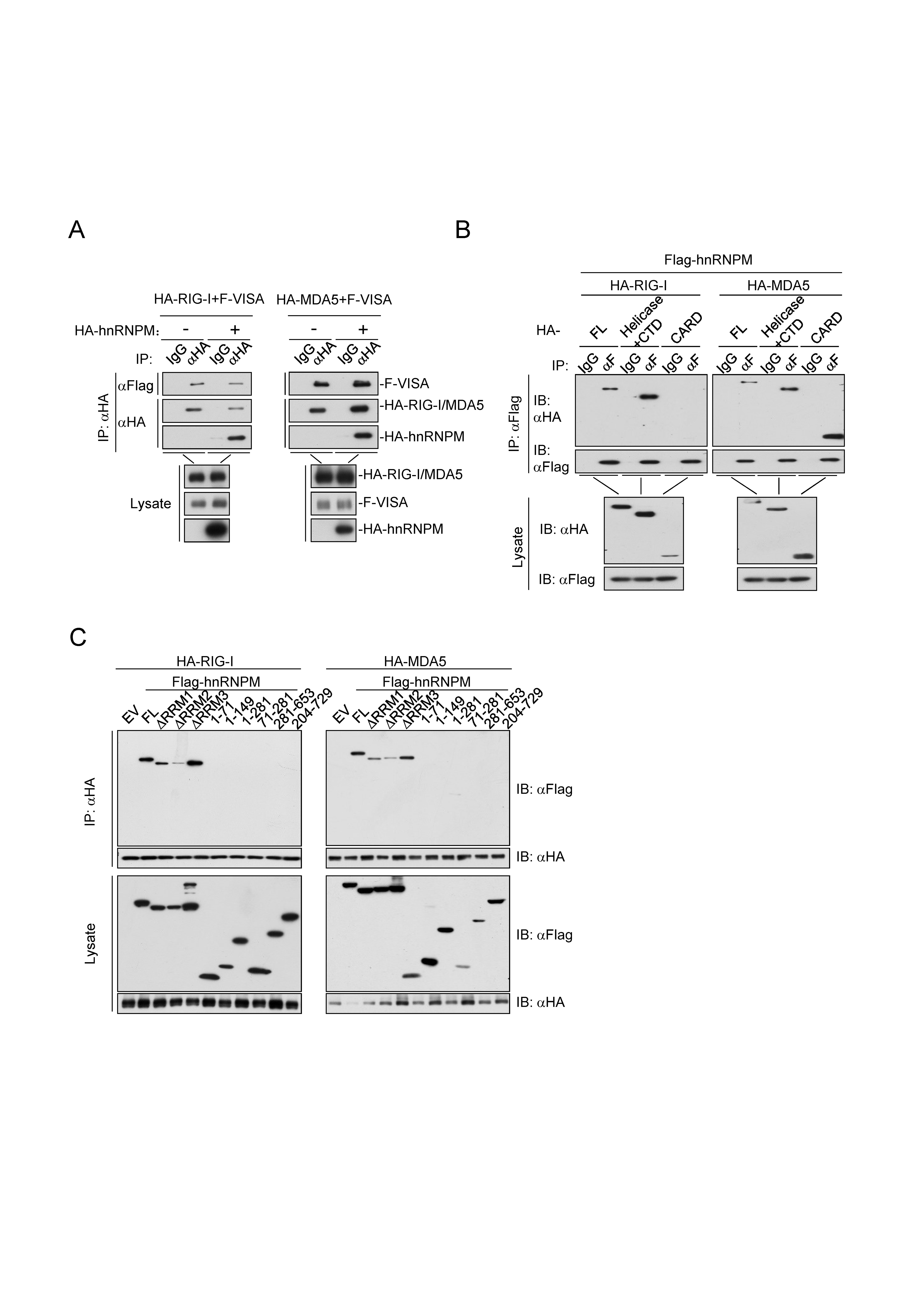

Supplement: S2 Fig — (A) hnRNPM had no effect on the interaction of RLR with their adaptor. HEK293 cells were transfected with the indicated plasmids before co-immunoprecipitation and immunoblotting analysis with the indicated antibodies. (B&C) Domain mapping of the interactions between hnRNPM and RIG-I or MDA5.HEK293 cells were transfected with the indicated plasmids before co-immunoprecipitation and immunoblotting analysis with the indicated antibodies. The results were schematically presented in Fig 5D. FL, full length. (TIF) [file ppat.1007983.s002.tif]

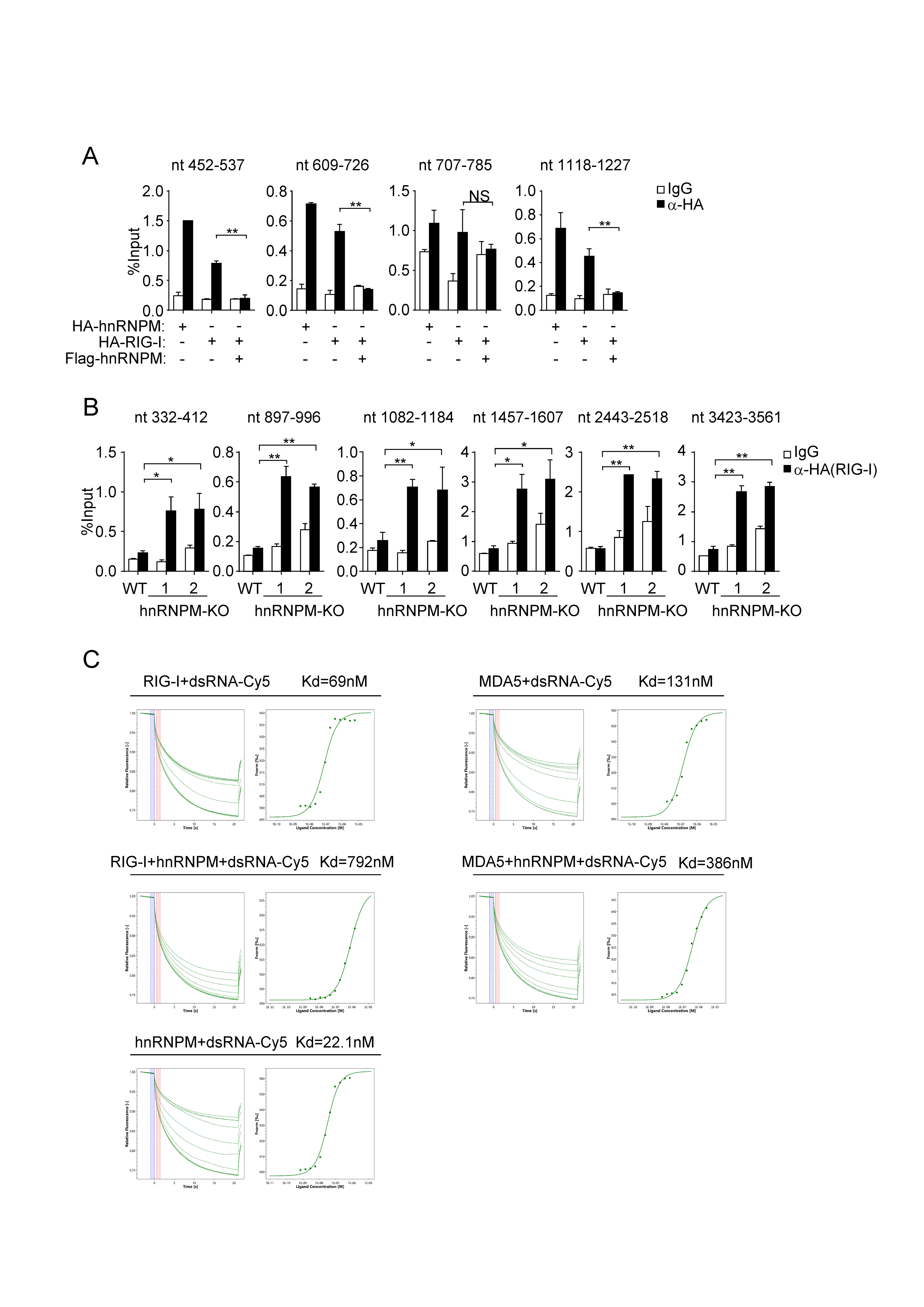

Supplement: S5 Fig — (A) Supplementary data for Fig 7A. (B) Supplementary data for Fig 7B. (C) Supplementary data to Fig 7D. *p < 0.05, **p < 0.01 (unpaired t test). (TIF) [file ppat.1007983.s005.tif]
