# Supplementary material for: The heterogeneous nuclear ribonucleoprotein hnRNPM inhibits RNA virus-triggered innate immunity by antagonizing RNA sensing of RIG-I-like receptors
Source: PLoS Pathog. 2019 Aug 21;15(8):e1007983. doi: 10.1371/journal.ppat.1007983 (PMC6703689; doi:10.1371/journal.ppat.1007983)
Supplement: S1 Table — The SeV genome primer sequences used in Q-PCR were described in the table. (DOC) [file ppat.1007983.s006.doc]

**Table S1. The Q-PCR primers for SeV genome**

| SeV RNA | Forword | Reverse |
| --- | --- | --- |
| nt56-144 | AGGGTCAAAGTATCCACCCTG | CGAAGGTGCTCAACAACCCG |
| nt64-197 | AGTATCCACCCTGAGGAGCA | AGCACCTCCTCCCGACTTAT |
| nt 101-228 | TTTGCTGCCAAAGTTCACGA | CTGAGACTGTGCTCCTCTGG |
| nt 127-230 | GGTTGTTGAGCACCTTCGAT | CACTGAGACTGTGCTCCTCT |
| nt 184-261 | CGGGAGGAGGTGCTGTTATC | CATCATCAGTCACACTTGGGC |
| nt 332-412 | AGGAGGGTTCCTCGTCTCTC | TCGGCGTTTACTCCGTTTGT |
| nt 397-519 | ACGGAGTAAACGCCGATGTC | GCCATTCTGTGGTCCTCTCA |
| nt 452-537 | GAGGACGAAGACAGACGGAT | TGACCCTAGGTCCAAACAGC |
| nt 609-726 | CCTGCATGCCTAGGAGCAAT | CGTCTTGTCTGAACGCCTCT |
| nt 707-785 | AGAGGCGTTCAGACAAGACG | AACCGAGCCTATCCCCTCAA |
| nt 897-996 | TACATCCGAGATGCAGGGCT | TATCGGGCCTCAGGTTTGAC |
| nt 1082-1184 | TGGTGAATTTGCTCCAGGCA | TGTCCTCCCTGTGACGTACT |
| nt 1457-1607 | TGAAAGATGGGCACGTCAGG | GCAGAATCCTCTTGCCGTCT |
| nt 1117-1225 | GGAGTTACGCCATGGGAGTC | GCCACGGCTTGTCCTAGTAA |
| nt 1753-1839 | CATCCACCGATCGGCTCAGG | TAAGTGTAGCCGAAGCCGTG |
| nt 1962-2081 | CAACTGACATCGGAGGGGAC | GGTCGACGGTGTTGAGACTT |
| nt 1821-1961 | ACGGCTTCGGCTACACTTAC | GTTCACTCGACAGGACAGCA |
| nt 2443-2518 | TGGCAGCTCACATAGTGCAA | CCGTAGCACAGCCTCTTCAA |
| nt 2532-2674 | GACCTACCAACAGTGGGTCC | GTCCCCAGAGTTGATGTGCT |
| nt 2898-2995 | ATGTGTTTGCAAGACGTGCC | ACGAGCGGAAGATTTCTCGG |
| nt 3178-3293 | AAGGTCCCCCTCCGTTTTTG | CCTCTCGGATTAGGTCCGGT |
| nt 3423-3561 | AGAGCAGTCCCCTAAGCAGA | TCACCCGGGATCTAGTTGGT |
| nt 3885-4013 | GACTTGACAGAGCCGACCAG | TCCTCCTCACCGTAATTCTG |
| nt 3972-4071 | CTCTTAAAGGCCTGCACCGA | GGAGTGGAGCACCAATCGAA |
| nt 9568-9656 | TTGTGGAGTCGTTACTCGCC | GGGGTGGCCAAATGTCCTAA |
| nt 12213-12309 | GATGAAAGGTCGGAAGCCCA | CGTAGGCCCACGTATACACC |
| nt 13720-13823 | CTAGAGCCCCTTTTTGGGGG | CCAACGCCTGATCTGCCTAT |
| nt 14082-14185 | TATCCTGCTGAGGTGGCACT | TCCATGTCGAGCCAGGATTC |
